# Retraction: HER2 decreases drug sensitivity of ovarian cancer cells via inducing stem cell-like property in an NFκB-dependent way

**DOI:** 10.1042/BSR20180829_RET

**Published:** 2025-08-13

This article is being retracted from Bioscience Reports at the request of the Editor-in-Chief and the Editorial Board. This follows the receipt of a notification from a reader, alerting the Editorial Board to similarities between the Figure 1E HER2 OE panel and the Figure 1F IL-6 panel from a previous publication by Liu et al, 2018 (DOI: 10.1158/0008-5472.can-17-2761) with colour and scalebar modifications. Additionally, the Editorial Office has concerns surrounding similarities in the histological appearance of the Figure 6A + and +++ panels. The authors were contacted regarding these concerns and whilst they stated their intent to investigate, they have not since responded with any data, explanations or in response to our notice of retraction. Given the extent of the issues raised, the Editorial Board stands by the decision to retract the article.

